# Effect of Terminal Groups on Thermomechanical and Dielectric Properties of Silica–Epoxy Composite Modified by Hyperbranched Polyester

**DOI:** 10.3390/polym13152451

**Published:** 2021-07-26

**Authors:** Jianwen Zhang, Dongwei Wang, Lujia Wang, Wanwan Zuo, Lijun Zhou, Xue Hu, Dingyu Bao

**Affiliations:** 1 School of Electrical and Power Engineering, China University of Mining and Technology, Xuzhou 211116, China; jwzhang@cumt.edu.cn (J.Z.); dongwei.wang@cumt.edu.cn (D.W.); zuoww@cumt.edu.cn (W.Z.); huxue@cumt.edu.cn (X.H.); bdy189056603142021@163.com (D.B.); 2 State Key Laboratory of Internet of Things for Smart City, University of Macau, Macau 999078, China; 3 School of Electrical Engineering, Southwest Jiaotong University, Chengdu 611756, China; ljzhou10@163.com

**Keywords:** silica–epoxy composite, hyperbranched polyester, terminal groups, molecular dynamics, thermomechanical properties, dielectric properties

## Abstract

To study the effect of hyperbranched polyester with different kinds of terminal groups on the thermomechanical and dielectric properties of silica–epoxy resin composite, a molecular dynamics simulation method was utilized. Pure epoxy resin and four groups of silica–epoxy resin composites were established, where the silica surface was hydrogenated, grafted with silane coupling agents, and grafted with hyperbranched polyester with terminal carboxyl and terminal hydroxyl, respectively. Then the thermal conductivity, glass transition temperature, elastic modulus, dielectric constant, free volume fraction, mean square displacement, hydrogen bonds, and binding energy of the five models were calculated. The results showed that the hyperbranched polyester significantly improved the thermomechanical and dielectric properties of the silica–epoxy composites compared with other surface treatments, and the terminal groups had an obvious effect on the enhancement effect. Among them, epoxy composite modified by the hyperbranched polyester with terminal carboxy exhibited the best thermomechanical properties and lowest dielectric constant. Our analysis of the microstructure found that the two systems grafted with hyperbranched polyester had a smaller free volume fraction (FFV) and mean square displacement (MSD), and the larger number of hydrogen bonds and greater binding energy, indicating that weaker strength of molecular segments motion and stronger interfacial bonding between silica and epoxy resin matrix were the reasons for the enhancement of the thermomechanical and dielectric properties.

## 1. Introduction

The light weight of electric traction systems is a promising trend in the development of high-speed trains, which can effectively reduce the costs in operation and maintenance even track loads. However, the oil-immersed transformer, as the heaviest single device in the electric traction system, greatly limits the realization of light weight [1]. Dry-type transformers have lighter weight due to the removal of oil tanks and heat sinks, which are expected to replace oil-immersed transformers to achieve the goal of light weight.

Epoxy resin (EP) is widely used in dry-type transformers for its excellent insulation properties and chemical stability [2,3]. However, compared with the common dry-type transformers, the vibration phenomenon of dry-type transformers applied in high-speed train is prone to occur when the train is running at high speed, aggravating the fatigue damage of epoxy resin, which has higher requirements on the mechanical properties of epoxy resin for dry-type transformers applied in high-speed train [4]. In addition, the poor thermal conductivity of the epoxy resin cannot meet the heat-dissipation requirements for the dry-type transformer in a small vehicle body, which further aggravates the degradation of the mechanical properties of the epoxy resin. Therefore, the epoxy resin in the dry-type transformer applied high-speed train needs higher thermomechanical properties to adapt to the actual working conditions while maintaining dielectric properties.

Nanomaterials have some unique properties, such as small size effect, quantum effect, and surface effect, which have been gradually used in dielectric modification [4,5,6,7]. Researches have shown that the doping of nanoparticles can combine the excellent properties of the filler itself, such as high toughness and thermal conductivity, with epoxy resin to enhance the thermal, dielectric, and mechanical properties of epoxy composites [8,9,10,11,12,13]. However, the compatibility between nanoparticles and epoxy resin matrix is poor due to the high surface energy of filler, which often leads to agglomeration during doping process if there are not any surface treatments, failing to achieve the expected result [14,15]. Chemical modification on nanoparticles surface is an effective method to improve the dispersion of fillers in epoxy resin host and interface bonding between the two [16,17,18].

Hyperbranched polyester is a kind of highly branched three-dimensional dendritic polyester which can be used as an effective surface modifier to control the inorganic–organic interface between filler and polymer matrix [15,19,20]. Many active terminal functional groups of hyperbranched polyester grafted on nanoparticles can interact closely with the polymer matrix, acting as a bridge between the two interfaces, which leads to better dispersibility of fillers and interaction bonding to improve the performance of composite [21,22,23,24]. However, many studies concentrate on the traditional experimental tests, which have difficulty articulating internal mechanisms clearly. Notably, there are still some deficiencies in the research of hyperbranched polyester modification mechanisms of epoxy resin from the microscopic point of view, especially for the research on the relationship between the microstructure and performance of epoxy resins modified by hyperbranched polyester with different terminal groups.

In recent years, with the development of high-performance computing technology, molecular dynamics (MD) simulation has been widely used in the study of the microstructure and macro-properties of composite polymer materials [25,26]. MD simulation can build materials on the atomic scale, which greatly reduces the manufacturing cost and development cycle and then simulates the structure and behavior of molecules to calculate the properties of the materials [27,28]. At present, many studies have been conducted on the properties of nanocomposite epoxy resins, using MD simulation [29,30,31,32]. Du et al. studied the effects of different grafting density of amino silane coupling agents on thermomechanical properties of crosslinked epoxy resin. The results showed that, with the increase of the grafting density, the mechanical properties and glass transition temperature of epoxy resin showed a trend of increasing first and then decreasing [33]. Jeyranpour et al. investigated the effect of the type of curing agent on the thermomechanical properties of epoxy resin, and DGEBA/TETA and DGEBA/DETDA epoxy systems were built, respectively, using MD simulation. The results showed that, under the same crosslinking density, the DGEBA/DETDA had a higher glass transition temperature than that of DGEBA/TETA, while higher mechanical properties were observed in the case of DGEBA/TETA [34]. Xie et al. studied the effects of size of silica and crosslinking density on the microstructure and thermomechanical properties of silica–epoxy composite. The increase of crosslinking density enhanced the thermomechanical properties of epoxy resin. Meanwhile, the decrease of particle size also contributed to the improvement of thermomechanical properties [35]. Chang et al. investigated the effect of amino-modified silica on the corrosion resistance of epoxy resin, and better corrosion resistance was found when epoxy resin incorporated with silica modified by amino groups [36]. The abovementioned research studies proved that MD simulation is an essential instrument in terms of analyzing the relationship between the microstructure and performance of epoxy resin, which is also expected to provide new approaches for the design and preparation of epoxy composites with enhanced performance. Inspired by these research studies, MD simulation was applied to reveal the systematic and detailed interpretation of the relationship between thermomechanical and dielectric properties and structure of epoxy resin brought by hyperbranched polyester with different terminal groups.

In this paper, the effects of terminal groups on the thermomechanical and dielectric properties of epoxy resin composite incorporated with silica grafting with hyperbranched polyester were studied based on MD simulation. A group of pure epoxy resin and four groups of silica–epoxy resin composite were established, where the silica surface was hydrogenated, grafted with silane coupling agent (KH560 was selected), hyperbranched polyester with terminal carboxyl (CHBP), and hyperbranched polyester with terminal hydroxyl (HHBP), respectively. Moreover, the thermomechanical and dielectric properties were calculated quantitatively including thermal conductivity, glass transition temperature, elastic modulus, and dielectric constant. The four microscopic indexes such as mean square displacement, hydrogen bonds, fractional free volume and binding energy were computed to explain the internal modification mechanism from a microscopic view. This research laid a theoretical foundation for the preparation of high-performance epoxy resin.

## 2. Materials and Methods

The models in this paper were built using Material Studio software (Accelrys Co., Ltd., San Diego, CA, USA). Five group of models were established to investigate the influence of terminal groups on the performance of silica–epoxy resin modified by hyperbranched polyester. The models were defined as follows: pure epoxy resin (EP), epoxy resin composite model doped into silica (SiO_2_-EP), epoxy resin composite model doped into silica grafted silane coupling agent KH560 (SiO_2_-KH560/EP), epoxy resin composite model doped into silica grafted with hyperbranched polyester with carboxyl terminal group (SiO_2_-CHBP/EP) and the epoxy resin composite model doped into silica grafted with hyperbranched polyester with hydroxyl terminal group (SiO_2_-HHBP/EP). The specific steps of model construction were as follows.

### 2.1. Epoxy Resin Models

**Figure 1:**
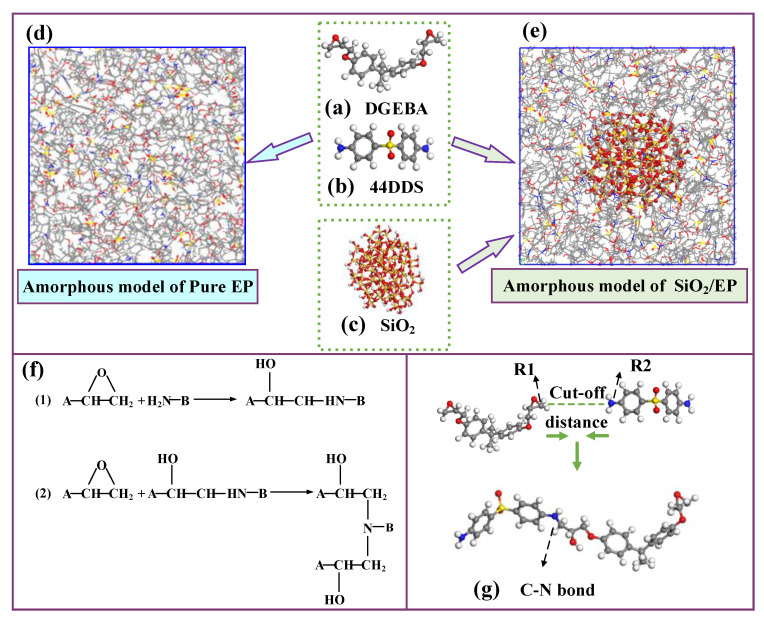
(**a**) Molecular models of epoxy resin host, (**b**) molecular models curing agent, (**c**) silica nanoparticle, (**d**) amorphous model of pure EP, (**e**) amorphous model of epoxy containing silica, (**f**) chemical reactions of crosslinking, and (**g**) C-N bond.

Bisphenol A epoxy resin (DGEBA) and 4,4′-diaminodiphenyl sulfones were built to act as polymer host and the curing agent, respectively, as shown in Figure 1a,b. In the experimental study, the average degree of polymerization of DGEBA is usually between 0.1 and 0.2, so the polymerization of DGEBA molecules was set to zero in this simulation [37]. Reference [32] indicates that the cell size had no large effect on the calculated properties. Considering the actual reaction situation that two DGEBA molecules react with one 44DDS to form a three-dimensional crosslinked epoxy resin, we constructed a 50 × 50 × 50 Å^3^ amorphous model containing DGEBA and 44DDS molecules by a 2:1 ratio, using the Amorphous Cell Tools, as shown in Figure 1d [38]. The initial density of the amorphous model was set to 0.6 g/cm^3^, and periodic boundary conditions were applied to the unit cell to avoid the size effect of the material.

An energy optimization calculation with energy convergence of 1 × 10^−4^ kcal/mol and displacement of 5 × 10^−4^ Å for 10,000 steps iterations was performed in the established amorphous model. Subsequently, the model was annealed in the constant volume and temperature (NVT) ensemble from 300 to 900 K to obtain the lowest energy conformation. Molecular dynamics simulations were conducted based on the lowest energy conformation. To reduce the stress generated in the modeling and reach the actual density of epoxy resin, MD simulations were first performed in the NVT ensemble for 500 ps at 300 K; and in the constant pressure and temperature (NPT) ensemble, the model was equilibrated for 1000 ps at 1 atm. In the abovementioned simulations, the force field COMPASS was applied, and the electrostatic and Vander Waals were calculated, using the Ewald and Atom Based, respectively. The Andersen and Berendsen were used to control temperature and pressure.

**Figure 2:**
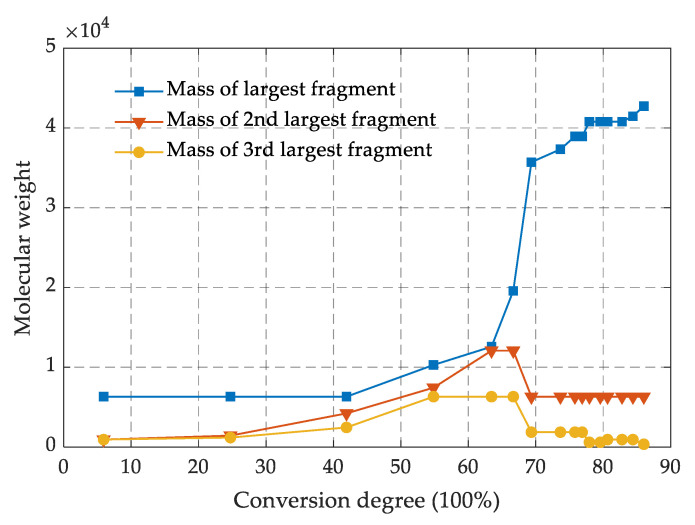
Molecular-weight variation of fragments with the conversion degree.

The crosslinking process between the epoxy resin monomer and curing agent monomer was simulated by running the PERL language in the next steps, and the involved chemical reactions are shown in Figure 1f. The carbon atom of epoxy group in DGEBA and the nitrogen atom of amino group in 44DDS were marked as R1 and R2, respectively. When the distance between the atom R1 and the atom R2 met the preset distance, a crosslinking reaction occurred to form a C-N bond. The target conversion degree (TCD) was set to 80%, the initial cutoff distance R_0_ was 3 Å, the maximum cutoff distance R_max_ was 8 Å and the crosslink temperature was set to 350 K. Figure 2 shows the changes of molecular weight of the first three largest fragments in the model as the crosslinking density increase. The molecular weight of the largest fragment gradually increased, indicating that the epoxy resin monomer and curing agent molecules were continuously reacting to form a whole crosslinked network. The second largest and the third largest fragment molecular weight first increased and then decreased, which was mainly attributed to fact that the original segments were also crosslinked with the largest segment as the crosslinking density approached its maximum.

Finally, the same energy optimization and molecular dynamics simulations were performed as before to obtain the crosslinked model.

### 2.2. Silica–Epoxy Resin Models

In this study, epoxy resin composites containing silica were treated with four different surface treatments: the silica surfaces were hydrogenated, grafted with silane coupling agent, hyperbranched polyester with terminal carboxyl, and hyperbranched polyester with terminal hydroxyl, respectively.

**Figure 3:**
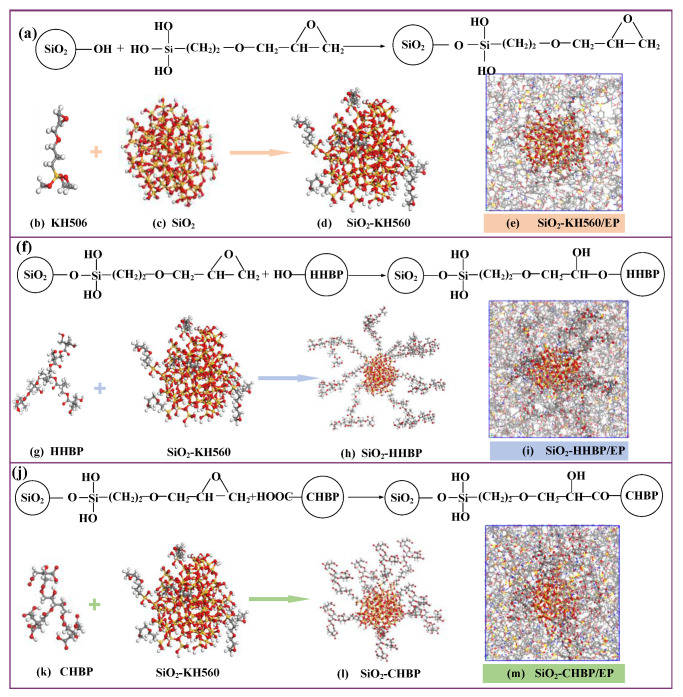
(**a**) Reactions of grafting KH560; (**b**) KH560; (**c**) silica particle; (**d**) silica particle with KH560; (**e**) epoxy composite containing silica particles grafted with KH560; (**f**) reactions of grafting HHBP; (**g**) HHBP; (**h**) silica particle grafted with HHBP; (**i**) epoxy composite containing silica particles grafted with HHBP; (**j**) reactions of grafting CHBP; (**k**) CHBP; (**l**) silica particle grafted with CHBP; (**m**) epoxy composite containing silica particles grafted with CHBP.

First, a spherical silica particle with a radius of 10 Å was constructed, and the unsaturated oxygen atoms and unsaturated silicon atoms on the silica surface were bonded with hydrogen atoms and hydroxyl to perform surface hydrogenation, respectively. Based on grafting mechanism shown in Figure 3, eight hydroxyl groups at different positions among eighty-two hydroxyl groups on the surface of established silica were randomly selected to manually graft the silane coupling agent and two kinds of hyperbranched polyesters with different terminal groups on the silica surface, with a target grafting rate of 10%.

The four constructed silica above were placed in the center of cells, and the crosslinking process and MD simulations were performed according to the methods in Section 2.1, where the TCD was also maintained at 90%. In addition, the volume fraction of silica in the four composites’ models was kept at about 7.2% by adjusting the molecular numbers of DGEBA and 44DDS.

### 2.3. Simulation Details

**Figure 4:**
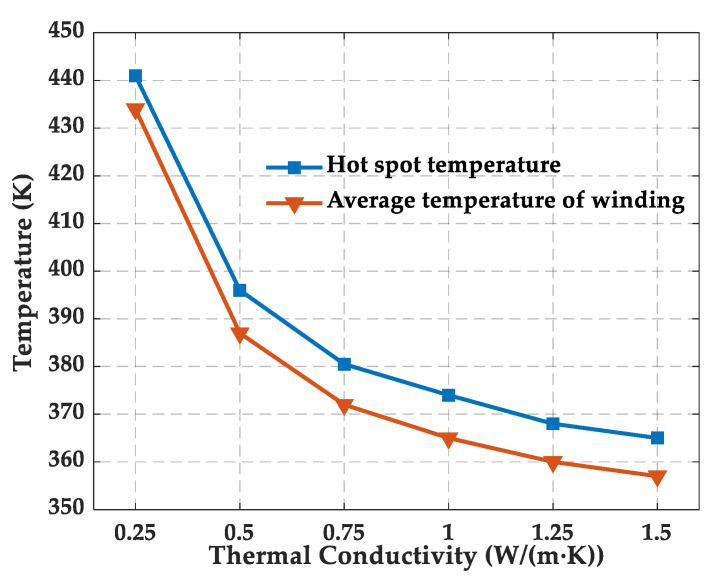
Change of hot-spot temperature and average temperature of winding with the thermal conductivity of epoxy resin.

The temperature rise can exacerbate the deterioration of epoxy resin performance. To determine the temperature range in the simulation, the temperature field of dry-type on-board traction transformer was simulated, and the hot-spot temperature and average temperature of the winding were obtained under different thermal conductivities of the epoxy resin, as shown in Figure 4.

The hot-spot temperature of the winding of could reach 441 K when the ambient temperature and thermal conductivity was set as 300 K and 0.25 W/(m·K), respectively, so the maximum temperature in the MD simulation should be higher than 441 K, considering the rise in ambient temperature and the low thermal conductivity of epoxy resin (usually below 0.2 W/(m·K)). In addition, the glass transition temperature was calculated by fitting the density–temperature curve in the temperature interval above and below the glass transition temperature, which is generally from 400 to 500 K, so the maximum temperature was chosen to be 600 K. Considering the heat generated by the transformer during actual operation, the epoxy is usually exposed to a minimum temperature higher than room temperature, so the minimum temperature of 300 K was chosen. Therefore, in this paper, the variation in the performance of the epoxy resin was studied from 300 to 600 K.

Based on the crosslinked models established in Section 2.1 and Section 2.2, the quasi-static cooling process was performed. First, MD simulations were conducted successively for 500 ps in NVT and NPT ensemble in 600 K, and the simulation parameters were consistent with Section 2.1. Then, the crosslinked models were cooled from 600 to 300 K, at a cooling rate of 25 K/1000 ps [31]. Based on this process, the structure parameters of epoxy resin at different temperatures were obtained which lay the foundation for the subsequent analysis of performance.

## 3. Results and Discussion

### 3.1. Glass Transition Temperature

The glass transition temperature (*T*g) is the corresponding temperature when the polymer undergoes a phase change from the glassy to the rubbery state, which determines the upper temperature limit for the normal operation of material. This transformation process will lead to deterioration of various properties of the epoxy resin, such as thermomechanical properties and degradation stability. Therefore, the enhancement of glass transition temperature is of great significance to improve the thermomechanical properties of epoxy resin. Around the *T*g, the polymer shows an abrupt change in density and volume at the macroscopic level and presents a shift from a cohesive to a kinematic state of the molecular segments microscopically as well. Therefore, the *T*g was calculated by combining the mean square displacement (MSD) and the density–temperature curve in this simulation. The expression for MSD in a system containing *N* atoms is as follows:

(1) $$\mathrm{MSD}=\frac{1}{3N}\overset{N-1}{\underset{i=0}{\sum }}\left({\left|R_{i}{(t)\;-\;R}_{i}\left(0\right)\right|}^{2}\right)$$

where *R_i_*(*t*) and *R_i_*(0) are the displacement vectors of atom *i* at time *t* and initial time. The MSD quantitatively characterizes the strength of the polymer segments motion. When polymers experience glass transition, the torsional and rotational motion of the molecules combined with the local motion can induce abrupt change of segments motion, which is manifested as an abrupt jump in the MSD curve in a specific temperature range. Therefore, the glass transition region can be roughly estimated by finding a larger gap in the MSD–time curves. Then, the density–temperature scatter chart is linear fitted for the range below and above the candidate temperature interval, and *T*g is obtained from the intersection of the two fitted lines [35].

**Figure 5:**
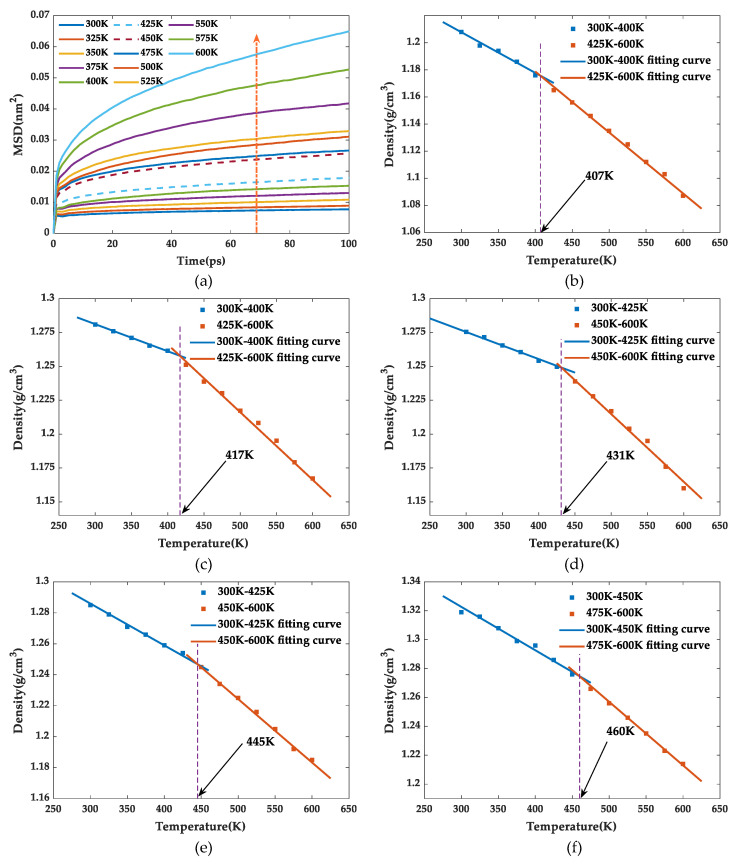
(**a**) The MSD–time curves of epoxy resins; (**b**) density curves versus temperature of pure EP; (**c**) density curves versus temperature of SiO_2_/EP; (**d**) density curves versus temperature of SiO_2_-KH560/EP; (**e**) density curves versus temperature of SiO_2_-HHBP/EP; (**f**) density curves versus temperature of SiO_2_-CHBP/EP.

Taking SiO_2_-HHBP/EP as an example, the MSD curve for the first 100 ps under the NPT system ensemble was shown in Figure 5a. The MSD curve showed a very significant change between 425 and 450 K which was defined as the candidate temperature interval. Then, the *T*g was obtained from the intersection point of fitting the density–temperature relationship curves between the temperature intervals of 300 and 425 K and of 450 and 600 K. The other models of *T*g were obtained using the same method.

Table 1 shows the *T*g from simulation and references. The difference between two results is mainly due to the difference in the types of epoxy resin matrix and curing agent. The *T*g of the pure epoxy resin was 407 K, which was lower than all silica–epoxy composites. Incorporated silica into epoxy matrix enhanced the *T*g to various degrees, where the enhancement effect was more obvious through chemical modification on silica surface. Compared with the pure epoxy resin, the *T*g of SiO_2_/EP, SiO_2_-KH560/EP, SiO_2_-HHBP/EP, and SiO_2_-CHBP/EP increased by 10, 24, 38, and 53 K, respectively, indicating that hyperbranched polyesters with terminal carboxy contributed most to the improvement of *T*g.

To further investigate the effect of the concentration of silica on the *T*g, an epoxy composite modified by HHBP with a volume fraction of 5% were constructed. The calculated results are shown in Table 1. The glass transition temperature increased with the increase of concentration of silica. This phenomenon suggests that the combination of the increase in silica concentration and grafting hyperbranched polyesters on its surface can effectively increase the glass transition temperature of epoxy resins.

### 3.2. Thermal Conductivity

Thermal conductivity is a key parameter to characterize the thermal performance of epoxy resin, which determines the ability of heat dissipation. In the actual operation, power equipment always faces huge heat accumulation, which aggravates deterioration in the thermal aging and mechanical properties of epoxy resin. Therefore, improving the thermal conductivity is a key measure to enhance the stable working ability of epoxy resin.

**Figure 6:**
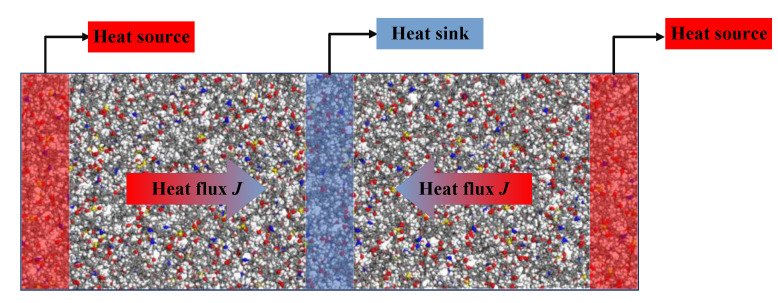
Schematic diagram of RNEMD model.

Many studies have been conducted to calculate the thermal conductivity of materials using molecular dynamics simulations. In this paper, reverse non-equilibrium molecular dynamics (RNEMD) was applied. Figure 6 presents the schematic of RNEMD. The basic idea of RNEMD is to apply a heat flux to the system and measure the induced temperature gradient. During the simulation process, the model was divided into several layers with equal width along the heat flux direction (40 layers in this simulation), and the model was expanded three times to facilitate the division of layers. The two side areas and middle areas of models were set as heat source and heat sink, respectively. Then the heat flux was generated by exchanging kinetic energy of the coldest atoms in heat source (with slowest speed) and the hottest atoms in source sink (with fastest speed).

Based on Fourier’s law of heat transfer, the thermal conductivity (*λ*) was calculated based on Equation (2):

(2) $$\lambda =\frac{J}{\nabla T}\;\;$$

where *J* is the heat flux and ∇T is the temperature gradient along the heat flux direction. The heat flux and temperature were calculated based on Equation (3) and Equation (4), respectively:

(3) $$J=\frac{\frac{1}{2}\sum_{i=1}^{N}\left(m_{1}v_{1}-m_{2}v_{2}\right)}{2\Delta tLA}\;$$

(4) $$T\left(z\right)=\frac{1}{3Nk_{B}}\overset{N}{\underset{i=1}{\sum }}m_{i}v_{i}$$

where *N* is the number of atoms for kinetic energy exchange; *m* and *v* are the mass and velocity of atoms; subscripts 1 and 2 indicate the hottest and coldest atoms, respectively; Δ*t* is the time interval at which the heat flux was applied; and *L* and *A* are the length and cross-sectional area of the model along the heat flux direction, respectively. Moreover, *k_B_* is the Boltzmann constant. The temperature gradient was derived by linearly fitting the temperature–distance relationship along the heat-flux direction.

**Figure 7:**
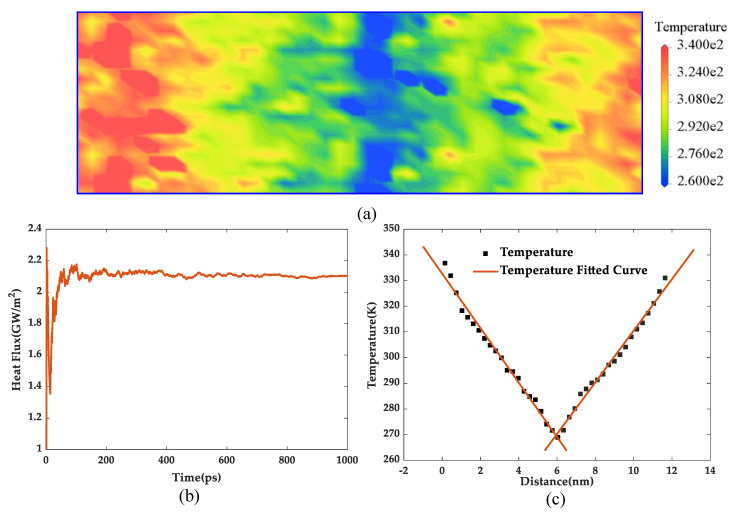
(**a**) Temperature distribution along the heat flux direction, (**b**) heat flux with the change of simulation time, and (**c**) temperature gradient diagram.

Taking the pure epoxy resin as an example, the stabilized heat flux and temperature distribution are shown in Figure 7. The heat flux was 2.102 GW/m^2^, the temperature gradient was 10.214 GK/m, and the thermal conductivity was calculated as 0.2147 W/(m·K) according to Equation (1). The calculated results of the thermal conductivity of the five models are shown in Table 2.

From the statistical results, the thermal conductivity of all models increased with the increasing of temperature. Pure epoxy resin had the lowest thermal conductivity of 0.2147 W/(m·K) at 300 K, and this value was slightly higher than the experimental result (0.21 W/(m·K)) reported in Reference [39]. The doping of pristine silica into epoxy resin can improve the thermal conductivity to 0.2641 W/(m·K) with a small increase ratio of 23.1%. The doping of pristine silica into epoxy resin can improve the thermal conductivity to 0.2641 W/(m·K) with a small increase ratio of 23.1%, which was slightly higher than the experimental result (0.2512 W/(m·K)) in Reference [40]. Silica itself has a high thermal conductivity, but direct addition had poor effectiveness mainly due to interfacial thermal resistance between silica and epoxy resin, which hindered seriously thermal transfer. In contrast, all epoxy composites containing silica with surface modifications had higher thermal conductivity, suggesting a reduction in the interfacial thermal resistance. Among different modification treatments, hyperbranched polyester brought an obvious increase effect, where hyperbranched polyester with terminal carboxyl had the most significant effectiveness. For example, at 300 K, the thermal conductivity was enhanced by 119.47%, higher than the 94.27% and 60.32% brought by the silane coupling agent and the hyperbranched polyester with terminal hydroxyl, respectively. From a microscopic point of view, the hyperbranched polyester with terminal carboxyl strengthened the interfacial bonding between silica and epoxy resin, which contributed to the overlap of their vibrational density of states, which increased the interfacial heat-transfer efficiency, leading to a decrease of interfacial thermal resistance and thermal conductivity enhanced accordingly [41]. In addition, the thermal conductivity increased as the concentration of silica increased (Table 2), which is mainly attributed to the fact that the increase of concentration contributed to the formation of more thermally conductive channels.

### 3.3. Mechanical Properties

In this paper, the mechanical properties were calculated by using the static constant strain method by applying a small strain within the elastic limit in an already energy equilibrated system, i.e., uniaxial tensile and compressive deformations in the *x*, *y*, and *z* axes and shear deformations in the *xy*, *xz*, and *yz* planes. After each application of strain, the energy optimization was re-conducted. The second-order derivative of potential energy with respect to strain was used to find the elastic matrix elements:

(5) $$C_{ij}=\frac{1}{V}\cdot \frac{\partial^{2}U}{\partial \varepsilon_{i}\partial \varepsilon_{j}}=\frac{\partial \sigma_{i}}{\partial \varepsilon_{j}}$$

where *U* is the potential energy, *σ* is the stress, and *ε* is the strain. In the simulation, the epoxy resin was assumed as isotropic material, and thus the stiffness matrix was simplified as follows:

(6) $$C_{ij}=\left[\begin{array}{cccccc} \lambda +2\mu & \lambda & \lambda & 0 & 0 & 0\\ \lambda & \lambda +\mu & \lambda & 0 & 0 & 0\\ \lambda & \lambda & \lambda +2\mu & 0 & 0 & 0\\ 0 & 0 & 0 & \mu & 0 & 0\\ 0 & 0 & 0 & 0 & \mu & 0\\ 0 & 0 & 0 & 0 & 0 & \mu \end{array}\right]$$

where *µ* and *λ* are the Lame elastic constants, which can be calculated based on Equation (7) and Equation (8):

(7) $$\lambda =\frac{1}{3}{(C}_{11\;}{+C}_{22}{+C}_{33})\;-\;\frac{2}{3}{(C}_{44}{+C}_{55}{+C}_{66})$$

(8) $$\mu =\frac{1}{3}{(C}_{44}{+C}_{55}{+C}_{66})$$

Therefore, Young’s modulus (*E*), bulk modulus (*K*), and shear modulus (*G*) were obtained according to the following equations:

(9) $$E=\mu \frac{3\lambda +2\mu }{\lambda +\mu }$$

(10) $$K=\lambda +\frac{2}{3}\mu$$

(11) G=μ

According to Equations (4)–(10), the mechanical property parameters of the five models were calculated in the range of 300–600 K. Table 3 compares the mechanical properties of pure epoxy from simulation with results from the previous references. From the statistical results, the results in simulation are close to those of the previous literature, indicating that the results of the calculations in this paper are equally reliable.

**Figure 8:**
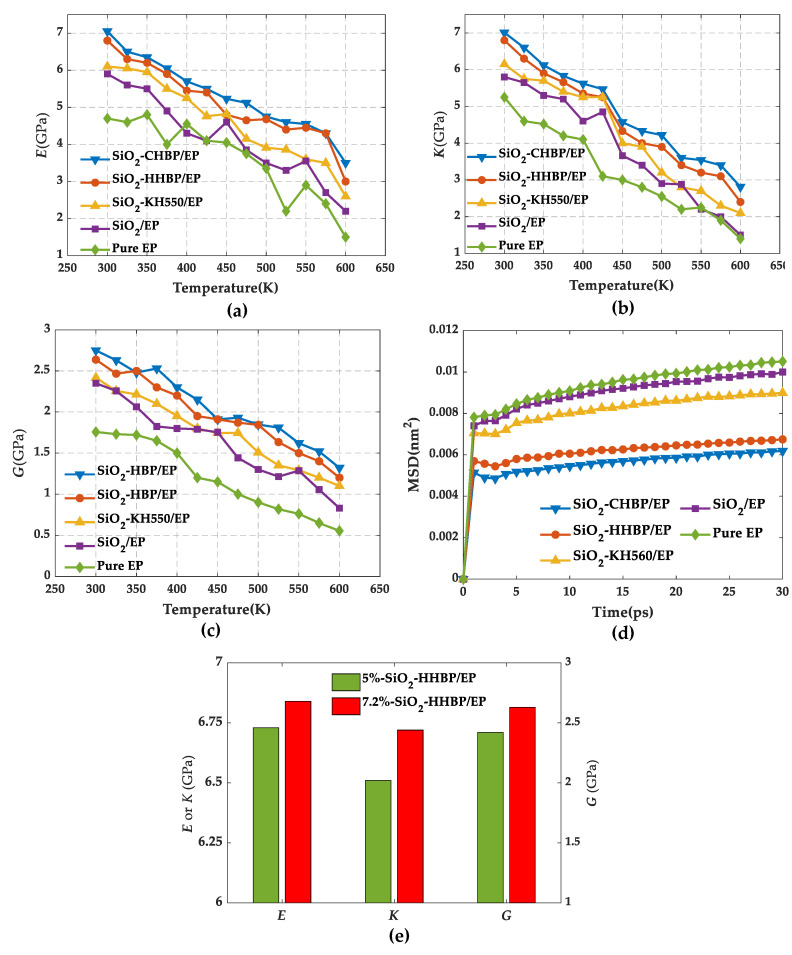
Mechanical properties under different temperatures. (**a**) Young’s modulus, (**b**) bulk modulus, (**c)** shear modulus, (**d**) mean square displacement of five models, and (**e**) mechanical properties of models under different concentration of silica.

As can be seen from Figure 8, the Young’s modulus, bulk modulus, and shear modulus fluctuated with a decreasing trend as the temperature increased, which was consistent with previous reports [38]. Comparing the five groups of models, the three moduli of pure epoxy resin were always the lowest at the same temperature, indicating that the doping of silica had improvement on the mechanical properties, and this effectiveness was also influenced by silica surface treatments. It can be clearly seen that, among the four groups of epoxy composites, grafting hyperbranched polyester with terminal carboxy on the silica surface showed the best mechanical properties. At 300 K, the Young’s modulus, bulk modulus, and shear modulus increased by 50.23%, 33.52%, and 54.78%, respectively. Meanwhile, the mechanical properties of epoxy composite with the volume fraction of 7.2% was better than those of epoxy composite with the volume fraction of 5%, as shown in Figure 8e.

On the one hand, silica with large modulus played a good supporting role when it was added into the epoxy resin matrix, so that epoxy composite was not easily deformed by external forces. Moreover, this enhancement is even more significant with the increase of the concentration of silica. On the other hand, surface modifications brought a strong interfacial interaction between silica and epoxy resin, and this interaction reached the highest value after grafting hyperbranched polyester with terminal carboxyl. The strong interaction has a pegging effect that anchors and entangles polymer segments on the nanoparticle surface, further limiting the movement of molecular chain segments. In addition, the thicker interfacial energy resulting from the strong interaction acts as a source of energy dissipation, which can absorb more energy and improve the mechanical properties of the material.

Furthermore, the MSD curves for five groups of models were computed at 300 K as shown as Figure 8d. It can be observed that the pure epoxy resin had the largest MSD value, indicating the strongest degree of segments movement within the system, while MSD presented the lowest value after the silica was grafted with hyperbranched polyester with terminal carboxy, resulting more stable structure and thus the mechanical properties presented superior. Furthermore, the analysis of the MSD values of the other models and their mechanical properties remained consistent.

### 3.4. Dielectric Properties

The large permittivity of the insulating material would intensify the accumulation of surface charge, which can easily lead to partial discharge; thus, it is meaningful to reduce the permittivity. In this paper, the permittivity of each model were calculated by using the total dipole moment formula, as shown in Equation (12) [30]:

(12) $$\varepsilon =1+\frac{\langle M^{2}\rangle \;{-\;\langle M\rangle }^{2}}{3\langle V\rangle k_{B}{T\varepsilon }_{0}}$$

where *M* is the dipole moment of each frame’s dynamic conformation, $\langle M^{2}\rangle$ is the average value of the dipole moment squared, ${\langle M\rangle }^{2}$ is the square of the mean dipole moment values, 〈V〉 is the volume of model, *k_B_* is the Boltzmann constant, *T* is the temperature, and *ε*_0_ is the vacuum permittivity. In addition, the combination of Maxwell Garnet theory and the total dipole moment formula provides a useful method to calculate the permittivity of modified nanoparticles [44].

The specific simulation method for the models was dynamic simulation under NVT ensemble at 300 K for 500 ps. The permittivity at 300 K was calculated by referring to Equation (12) after counting the dipole moment (*M*) of each frame’s dynamic conformation. Table 4 reflects the fluctuation of the dipole moments and permittivity.

From the statistical results, the pure EP had a permittivity of 3.32, which is close to the calculated results of 2.9 in Reference [45]. The permittivity of four epoxy composites were ranked as SiO_2_/EP > SiO_2_-KH560/EP > SiO_2_-HHBP/EP > SiO_2_-CHBP/EP. Epoxy composite modified by hyperbranched polyester with terminal carboxyl on the silica surface had the lowest dielectric constant of 2.45, with a decrease ratio of 26.2%, compared with pure EP. Silica had a strong adsorption of charge which inhibited the charge transfer and the fluctuation of the dipole moment, which guaranteed that the material was not easily polarized; thus, permittivity dropped correspondingly. Moreover, this adsorption enhanced with the increase of the concentration, so the increase in the concentration of the silica contributed to a lower dielectric constant of epoxy resin. In addition, the interface between silica and epoxy resin matrix plays an important role in the permittivity. Hyperbranched polyester with terminal carboxy enhanced the interfacial bonding, which made it more difficult for polar polymer segments to steer. The abovementioned results indicated that grafting hyperbranched polyesters on silica were effective in reducing the permittivity of epoxy resin.

### 3.5. Hydrogen Bonds

**Figure 9:**
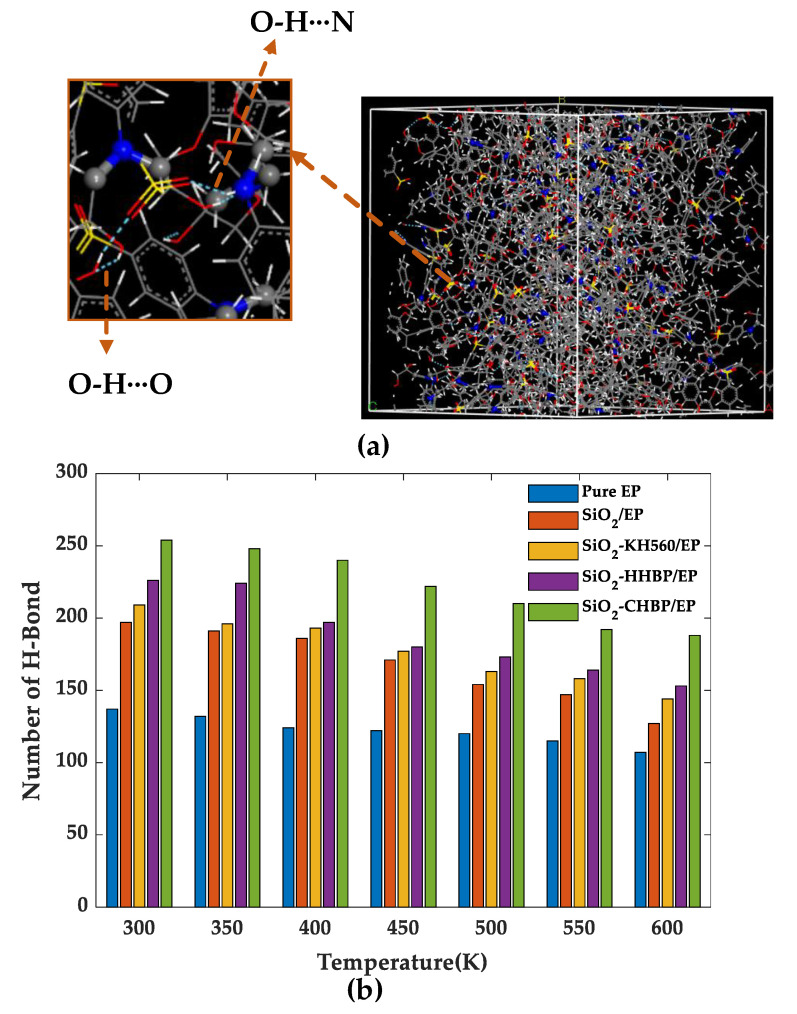
(**a**) Schematic diagram of different kinds of hydrogen bonds; (**b**) number of hydrogen bonds of five models.

The hydrogen bond is an interaction formed between two highly electronegative atoms through hydrogen atoms as the media, which plays an important role in the mechanical properties and aging resistance of polymers [23]. Furthermore, it can be expressed as X-H···Y, where X and Y are usually denoted as the donor and acceptor atoms. In this simulation, the hydrogen bonds formed between oxygen, nitrogen, and sulfur atoms were calculated. Furthermore, the geometric principle was applied to define hydrogen bonds. Figure 9 shows the schematic diagram of a hydrogen bonding in the model.

Figure 9 shows that the number of hydrogen bonds tended to decrease overall in the five models as the temperature increased, which is mainly attributed to the fact that the increased intermolecular distances caused by temperature rise broke the bonding conditions. The number of hydrogen bonds of the SiO_2_-HHBP/EP and SiO_2_-CHBP/EP was significantly higher than that of pure EP, SiO_2_/EP, and SiO_2_-KH560/EP under the same conditions. Among the two models of grafting the hyperbranched polyester on the silica surface, the SiO_2_-CHBP/EP had the most hydrogen bonds with increase ratio of 69.7% compared with pure epoxy resin at room temperature. The increase in hydrogen bonds produced stronger intermolecular interactions, which led to a tighter internal structure of the material, and the material had better mechanical properties and glass transition temperature than other models.

**Figure 10:**
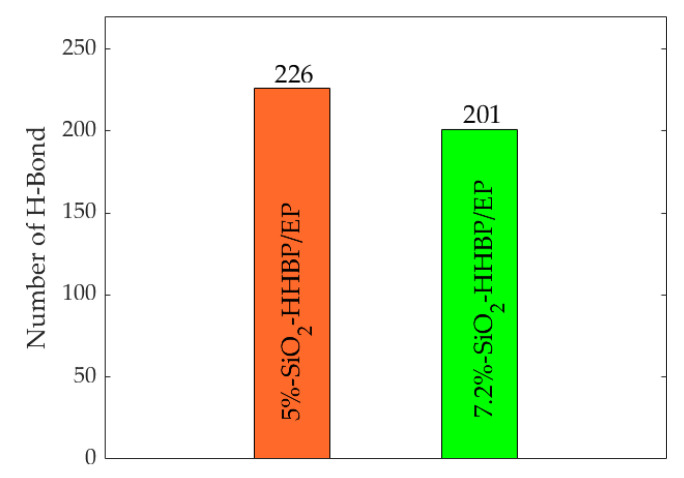
Number of hydrogen bonds of model under different concentration of silica.

In addition, the number of hydrogen bonds in epoxy composite under different concentration of silica were calculated as shown in Figure 10. The rise in concentration of silica increased the number of hydroxyl groups on its surface, leading to a strong interaction between the silica and the epoxy resin matrix, which resulted in more hydrogen bonds within the system. More hydrogen bonds will strengthen the intermolecular interactions and reduce the movement of the molecular segments, and the mechanical properties of the epoxy resin are correspondingly improved.

### 3.6. Free Volume Fraction

The free volume theory asserts that the volume of polymer consists of two parts: the volume of cavities between the molecular segments defined as free volume (*V_f_*) and the volume occupied by the segments (*V_o_*). The smaller free volume implies a decrease in the space for the potential movement of molecular segments, which has a positive effect on raising the glass transition temperature and the elastic modulus of the polymer. The relative free volume of a polymer is usually characterized by the free volume fraction, which is expressed as follows:

(13) $$\mathrm{FFV}=\frac{V_{f}}{V_{f}{+V}_{o}}$$

In this simulation, the free volume was calculated by creating Connolly surface, and the radius of the spherical probe was set to 1 Å. Table 5 reflects the free volume fractions of the five groups of models at 300 K. SiO_2_-CHBP/EP had the lowest free volume fraction, indicating the smallest space for the movement of molecular segments and the epoxy composite had the best thermomechanical properties, which was consistent with previous studies. Meanwhile, the FFV decreased with increasing concentration, which is mainly attributed to the fact that more silica occupied the original free space to reduce the molecular movement space. This result ultimately impeded the movement of the molecular segments, and the mechanical properties rose accordingly, which remained consistent with previous analyses.

**Figure 11:**
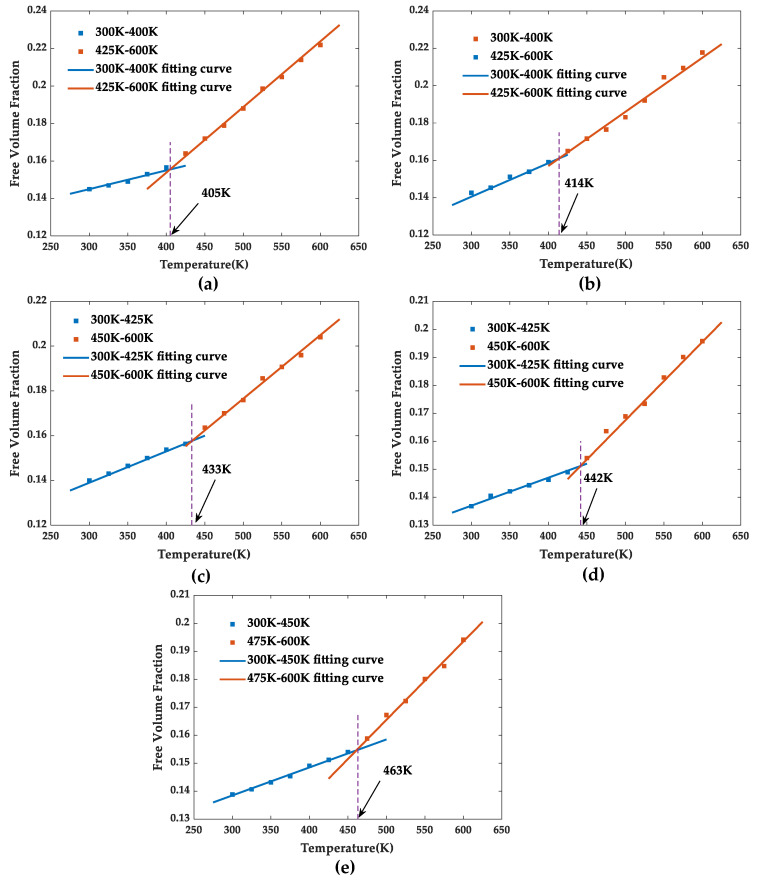
Free volume fractions of five models at different temperatures: (**a**) pure EP model, (**b**) SiO_2_/EP model, (**c**) SiO_2_-KH560/EP model, (**d**) SiO_2_–HHBP/EP model, and (**e**) SiO_2_–CHBP/EP model.

In addition, the motion degree of the molecular segments changes abruptly when polymers experience glass transformation and the corresponding motion space inevitably has an abrupt change; therefore, the glass transition temperature can also be simulated by fitting free volume fraction–temperature curve, as shown in Figure 11.The glass transition temperatures of pure EP, SiO_2_/EP, SiO_2_-KH560/EP, SiO_2_-HHBP/EP, and SiO_2_-CHBP/EP obtained by fitting the free volume–temperature curves were 405, 414, 432, 442, and 463 K, respectively. The results were nearly identical to the glass transition temperature obtained by the previously fitting density–temperature fitting curve.

### 3.7. Binding Energy

Researches have shown that the interfacial interaction between nanoparticles and polymer matrix is the key to the performance enhancement of nanocomposites. The binding energy quantitatively characterizes the strength of intermolecular interactions, and it was calculated according to Equation (14) [21]:

(14) $$E_{\mathrm{inter}}{=E}_{\mathrm{comp}}{-E}_{\mathrm{mat}}{-E}_{\mathrm{part}}$$

where *E*_inter_ is the bonding energy, *E*_comp_ is the non-bonding energy of the silica–epoxy composite, *E*_mat_ is the non-bonding energy of the epoxy resin matrix, and *E*_part_ is the non-bonding energy of silica.

In Table 6, the composite model modified with hyperbranched polyester exhibited stronger binding energy than that brought from silane coupling agent. Among two hyperbranched polyesters, SiO_2_-CHBP/EP had the highest binding energy, which can be attributed to the formation of more hydrogen bonds after grafting hyperbranched polyester with terminal carboxy. This means the polymer molecules can be tightly wrapped around silica, and, thus, the thermomechanical properties of epoxy composites were enhanced.

## 4. Conclusions

In this paper, the effect of terminal groups on thermomechanical and dielectric properties of silica–epoxy composite modified by hyperbranched polyester was investigated through molecular dynamics simulation. The following conclusions are drawn:

1 Incorporating silica into epoxy resin can improve thermomechanical and dielectric properties, and the improvement effect was the better when the silica surface was grafted with hyperbranched polyester, where the terminal group had an obvious influence on the improvement effect as well. The Tg of the SiO_2_-HHBP/EP and SiO_2_-CHBP/EP model increased by 38 and 53 K, and the thermal conductivity at 300 K increased to 0.4171 and 0.4825 W/(m·K)^−1^ with an increase ratio of 94.3% and 115.4%, respectively. The elastic modulus, including Young’s modulus, bulk modulus, and shear modulus, increased by 44.68%, 29.52%, and 50.08% and by 50.23%, 33.52%, and 54.78%. The permittivity was 2.62 and 2.45, with a decrease ratio of 21.1% and 26.2% compared with pure EP, respectively.
2 The analysis of the number of hydrogen bonds, mean square displacement, free volume fraction, and binding energy further clarified the mechanism of improvement. Among the five groups of models, epoxy resin incorporated with silica grafted with hyperbranched polyester with terminal carboxyl maintained the lowest MSD value and free volume fraction, verifying that the three-dimensional crosslinked structure of this system was the tightest. At the same time, the largest number of hydrogen bonds and the highest binding energy were found in the system. In general, grafting hyperbranched polyester proved to be an effective way to control and improve thermomechanical and dielectric properties of epoxy resins.

## Figures and Tables

**Table 1:** *T*g (units: K) of models.

Model	*T*g	*T*g in Reference
Pure EP	407 K	437 [29]
SiO_2_/EP	414 K	456 [30]
SiO_2_-KH560	431 K	
SiO_2_-HHBP/EP	445 K	
SiO_2_-CHBP/EP	460 K	
5%-SiO_2_-HHBP/EP	433 K	

**Table 2:** Thermal conductivity of models under different temperatures (units: W/(m·K)).

Temperature	Pure EP	SiO_2_/EP	SiO_2_-KH560/EP	SiO_2_-HHBP/EP	SiO_2_-CHBP/EP	5%-SiO_2_-HHBP/EP
300 K	0.2147	0.2641	0.3442	0.4171	0.4825	0.3745
400 K	0.2315	0.3026	0.3838	0.4517	0.5264	
500 K	0.2573	0.3308	0.4159	0.5045	0.5614	

**Table 3:** Mechanical properties of models under different concentration of silica (units: GPa).

Mechanical Properties	Results from Simulation	Results in References
*E*	4.84	5.21 [42] 4.36 [43]
*K*	5.21	4.61 [38]
*G*	1.76	1.64 [43]

**Table 4:** The fluctuation of the dipole moments and the permittivity of models.

Models	$\langle M^{2}\rangle \;-\;{\langle M\rangle }^{2}$	Permittivity
Pure EP	1571.3898	3.32
SiO_2_/EP	1338.5125	3.05
SiO_2_-KH560/EP	1246.2747	2.84
SiO_2_-HHBP/EP	1097.2636	2.62
SiO_2_-CHBP/EP	982.1186	2.45
5%-SiO_2_-HHBP/EP	1171.7691	2.73

**Table 5:** The free volume fractions of models (units: 100%).

Models	Free Volume Fraction
Pure EP	14.52
SiO_2_/EP	14.26
SiO_2_-KH560/EP	14.02
SiO_2_-HHBP/EP	13.68
SiO_2_-CHBP/EP	13.41
5%-SiO_2_-HHBP/EP	13.85

**Table 6:** The binding energy of five models (units: kcal/mol).

Models	Binding Energy
SiO_2_/EP	−231.14
SiO_2_-KH560/EP	−364.45
SiO_2_-HHBP/EP	−653.49
SiO_2_-CHBP/EP	−843.12

## Data Availability

Not applicable.
